# 6S management project to improve workplace productivity in Primary Health Centre in India

**DOI:** 10.1186/s12913-025-13379-0

**Published:** 2025-10-01

**Authors:** Poosa Sravanthi, Ravibabu Koppala, Subhodip Mitra, Naveen Kumar Pera

**Affiliations:** 1 Department of Hospital Administration, Kasturba Medical College, Manipal, Manipal Academy of Higher Education, Manipal, Karnataka India; 2 Department of Hospital Administration, Vydehi Institute of Medical Sciences, Whitefield, Bangalore, Karnataka India; 3 Department of Hospital Administration, All India Institute of Medical Sciences, Kalyani, West Bengal India

**Keywords:** 6S, Lean Healthcare, Primary Health Centre, India, Workplace Safety, Process Improvement, Quality Management

## Abstract

**Background:**

Primary Health Centers (PHCs) in India face challenges of overcrowding, infrastructure vows, and disarrayed workflows. The 6S management method—Sort, Set in Order, Shine, Standardize, Sustain, and Safety—offers a structured, low-cost approach to improving efficiency, safety, and quality in healthcare delivery.

**Aim:**

To examine the feasibility and impact of implementing the 6S method in a PHC setting, focusing on improvements in workplace organization, safety, and healthcare service processes.

**Methods:**

A prospective longitudinal study was conducted at a PHC in India over three months. The 6S method was introduced through staff by conducting training to them, weekly audits, and feedback mechanisms. Pre- and post-intervention assessments were carried out using two structured evaluation tools: one based on quantitative scoring, and another based on operational compliance. Data were analyzed using Jamovi software.

**Results:**

Overall workplace performance improved from approximately 30% before implementation to 95% after. The most significant gains were observed in Set in Order (5% to 100%), Shine (0% to 95%), and Standardize (20% to 100%). Evaluations also revealed increased adherence to safety protocols, improved cleanliness, reduced visual cluttering, and better staff discipline. A minor gap remained in achieving paperless standardization, but the transformation reflected a shift from a high-risk environment to a high-performing, lean healthcare facility.

**Conclusion:**

Implementation of the 6S method significantly improved workplace efficiency, hygiene, safety, and standardization in a resource-constrained PHC setting. The approach is feasible, scalable, and aligned with national healthcare quality improvement priorities.

**Supplementary Information:**

The online version contains supplementary material available at 10.1186/s12913-025-13379-0.

## Introduction

Efficient healthcare delivery depends not only on clinical excellence but also on operational discipline, workplace organization, and safety—all of which directly impact patient outcomes. This is especially relevant in resource-limited settings like India, where public healthcare institutions often face challenges such as cluttered workspaces, inconsistent practices, and hygiene issues [1]. PHC (Primary Health center) in India is foundational unit of public health delivery system in India with one medical officer, four nurses, 2 health workers, one pharmacist, on driver, three environmental health workers as manpower and catering to basic clinical services and laboratory services. They act as the first point of contact for a citizen to the medical system. In the state where this study was conducted, there are 2000 + PHCs and it is third highest state in number of rural PHCs.

In India, Narayanamurthy et al. [1] highlighted that public healthcare institutions often struggle with basic process flow clarity, unorganized physical environments, and lack of standardization. These issues are echoed in Suman and Prajapati's national study [2], which observed that healthcare setups, especially in smaller cities, lack readiness for Lean or 6S due to insufficient staff training and undefined workflows. Similarly, Bhat et al. [3] conducted a multi-site case study showing that feasibility hinges on managerial support, staff engagement, and visual process control.

To address these systemic inefficiencies, healthcare systems around the world have increasingly turned to management methodologies like Lean and Six Sigma. Originally developed in the manufacturing sector, Lean aims to eliminate waste and optimize processes, while Six Sigma focuses on minimizing variation and defects. The integration of these two frameworks—Lean Six Sigma (LSS)—has been shown to yield significant improvements in service delivery, particularly in hospitals and laboratories [2, 3].

A key subset of Lean methodology is the 5S approach: Sort, Set in Order, Shine, Standardize, and Sustain. In the healthcare setting, it has been expanded to 6S by adding Safety as a core component. Implementing 6S in clinical environments helps promote visual control, workplace discipline, and a culture of cleanliness and order [4]. To evaluate organizational readiness, Ajmera and Jain [4] employed a fuzzy interpretive structural modeling (FISM) technique. Their findings reinforced the role of leadership, communication, and training in ensuring 6S feasibility. Several studies, including those in Indian healthcare contexts, have demonstrated that 6S contributes to reducing waste, improving efficiency, and enhancing patient satisfaction. Bhat et al. [5] also emphasized that engagement at multiple levels—top management to floor staff—is essential for Lean success, especially in Indian healthcare. Singh et al. [6] used the fuzzy DEMATEL method to map implementation barriers in India and found infrastructural limitations, inconsistent communication, and cultural resistance as top barriers. Workplace organization is core to 6S.

Despite its benefits, the success of 6S in healthcare heavily depends on factors such as staff training, leadership support, and continuous monitoring. Cultural resistance and lack of standard operating procedures often hinder its sustainability in public health institutions [7]. Nonetheless, evidence from both global and Indian studies confirms that when these barriers are addressed, 6S can significantly improve safety, hygiene, and workflow efficiency [8, 9]. Swarnakar et al. [7] took this further using the best–worst method, ranking factors like top management involvement, staff empowerment, and training as critical enablers. Bhat and Jnanesh [8] and Gijo et al. [9] illustrated these enablers in action through Lean Six Sigma applications that reduced cycle time and improved patient registration services, respectively. Beyond feasibility, the real strength of 6S lies in the improvements it brings to operational efficiency, safety, and staff behavior. Numerous studies provide empirical backing for its positive impact, particularly in constrained settings.

The 6S management method, evolving from the foundation 5S of Lean philosophy, has proven to be a robust tool for process discipline, visual workplace organization, and staff-driven improvements in healthcare. Safety as the sixth element has elevated its relevance in infection-prone and high-touch environments like hospitals and PHCs.

This study aims to evaluate the implementation of the 6S management method in a Primary Health Centre (PHC) in India using a before-and-after audit approach. By comparing compliance and performance scores pre- and post-intervention, this research seeks to demonstrate the practical value of structured quality improvement tools in primary care settings.

## Materials and methods

### Study design and setting

This is a prospective, observational, and interventional study conducted over a three-month period in a public Primary Health Centre (PHC) located in India. The center reflects a low-resource healthcare environment with common systemic challenges such as limited infrastructure, high patient foot fall, and workforce constraints. Study Tool and Framework: The intervention was structured around the 6S management method: Sort, Set in Order, Shine, Standardize, Sustain, and Safety. Two validated evaluation tools were used to assess the pre- and post-implementation environment: A quantitative scoring tool with percentage-based compliance for each of the 6S pillars. A qualitative checklist based on binary (Yes/No) compliance across 25 operational indicators. Both tools were adapted for use in the PHC context and reviewed by subject experts to ensure relevance.

### Phases of the study

The study was conducted in three phases: Phase 1: Baseline Assessment: Initial evaluations were conducted using checklists based on American Society for Quality to assess the existing state of organization, cleanliness, safety, and standardization practices within the PHC [10, 11]. About 100 observations were made during different days by the author. Phase 2: Implementation of 6S: The 6S methodology was introduced through: Staff sensitization sessions using visual aids and demonstrations, Designation of 6S champions among existing staff, Weekly audits, feedback discussions, and peer learning, Improvements such as visual control boards, sorting zones, color coding, and safety signage were introduced without requiring high-cost inputs.

### Phase 3

#### Post-implementation assessment

After 8 weeks, the checklists were reapplied by the author to measure improvements across the six categories of 6S for a 4-week period, over 100 observation slots. All observations were recorded, and field notes were maintained to capture contextual challenges and enablers.

### Data collection and analysis

The checklist scores and compliance data were tabulated and analyzed using Jamovi statistical software. Descriptive statistics such as means and percentages were calculated to quantify improvements across the 6S dimensions.

### Ethical considerations

This quality improvement initiative was conducted with permission from the PHC medical officer. No patient-identifiable data was collected, and the study did not involve any direct clinical intervention. All healthcare staff were informed and involved voluntarily. The study has Institutional Ethics Committee approval.

## Results

During the phase of observations in the first month, the findings have been collected. Table 1 shows the data collected during this phase of the study.

**Table 1:** Table showing scores observed Pre-6S training (before) evaluation of Primary Health Center

6S CHECK LIST USED DATE: 01/06/2024 to 30/06/2024		Number of Problems			Rating			
		5 or more			0			
		3 to 4			1			
		2			2			
		1			3			
		None			4			
RATING								
		0	1	2	3	4	Score	
Category	Item							Comments
Sort (Organization)	Distinguish between what is needed and not needed						10	
	Unneeded equipment, tools, furniture, etc. are present					4		Excellent control—no unnecessary items cluttering the area. Keep it up
	Unneeded items are on walls, bulletin boards, etc			2				Some visual clutter present. Periodically review and remove outdated or irrelevant notices
	Items are present in aisleways, stairways, corners, etc	0						Major safety concern. Immediate clearing required to ensure unobstructed pathways and prevent accidents
	Unneeded inventory, supplies, parts, or materials are present					4		Well-maintained, only necessary inventory is stocked. Maintain periodic audits
	Safety hazards (water, oil, chemicals, machines) exist	0						Immediate action required—hazardous conditions detected. Implement strict controls for chemical and spill management
Set in Order (Orderliness)	A place for everything and everything in its place						1	
	Correct places for items are not obvious	0						Lack of visual cues (labels, floor markings). Implement signage and layout diagrams
	Items are not in there correct places		1					Minor misplacements observed—conduct regular checks and user training
	Aisleways, workstations, equipment locations are not indicated	0						No visible location indicators—essential for streamlined workflow. Recommend color-coded zones
	Items are not put away immediately after use	0						Poor discipline in item handling. Reinforce accountability for tool/equipment storage
	Height and quantity limits are not obvious	0						High risk of stacking/misuse. Use visual boards and indicators for stock and tool levels
Shine (Cleanliness)	Cleaning, and looking for ways to keep it clean and organized						0	
	Floors, walls, stairs, and surfaces are not free of dirt, oil and grease	0						Dirty floors and surfaces present hygiene and safety risks. Introduce a cleaning checklist
	Equipment is not kept clean and free of dirt, oil, and grease	0						Equipment maintenance is neglected—can lead to malfunction or accidents. Enforce cleaning after each shift
	Cleaning materials are not easily accessible	0						Staff may not be cleaning due to lack of access. Set up dedicated cleaning stations
	Lines, labels, signs, etc. are not clean and unbroken	0						Damaged labels can confuse and cause delays. Replace and protect visual guides
	Other cleaning problems (of any kind) are present	0						Deep cleaning and structured cleaning schedules are required. Conduct regular inspections
Standardize (Adherence)	Maintain and monitor the first three categories						4	
	Necessary information is not available	0						Workers lack basic visual instructions. Display SOPs and workflow charts clearly
	All standards are not known and visible	0						Standard operating procedures are not reinforced. Display charts and train workers
	Checklists don't exist for all cleaning and maintenance jobs			2				Some areas lack structured checklists. Develop standard formats and assign responsibilities
	All quantities and limits are not easily recognizable			2				Absence of labeling and storage indicators. Use kanban cards or limit signs
	Many items cant be located in 30 s	0						Time loss due to poor organization. Adopt 6S tagging and color codes
Sustain (Self-Discipline)	Stick to the rules						8	
	How many workers have not had 6'S training					4		Excellent—most workers have undergone 6S training. Maintain refreshers annually
	Performance of 6 S audit weekly once	0						No record of 6S evaluations. Schedule routine audits and document findings
	Personal belongings not neatly stored			2				Some clutter observed in personal storage. Reinforce clean desk policy
	The number of times job aids are not available or up to dated			2				Outdated guides lead to confusion. Review and revise job aids regularly
	Inspections of 6S audit weekly once	0						Critical gap. Establish a weekly inspection system with accountable leads
Safety							13	
	Are employees wearing suitable PPE required for their current work activity				3			Most workers observed wearing PPE. Continue training and spot-checks
	Walkways, access to safety equipment is clearly identified and unobstructed (hazards, obstacles)					4		Excellent—access routes and emergency equipment clearly marked and unobstructed
	Is the working environment suitable for the work in hand (lighting, air quality, temperature)		1					Some inadequacies in ambient conditions. Evaluate lighting and ventilation needs
	Are correct equipment/tools provided for the current work activity				3			Tools are mostly adequate. Reassess needs based on user feedback
	Tools, equipment, parts, WIP and PPE stored correctly and safely (appropriate height, location)			2				Some disorganization in tool storage. Use labeled racks and PPE lockers
	TOTAL						36	

The researcher observations were tabulated at various time intervals, on different days of the week, different shifts. The suggestions after the evaluation have been suggested later. Table 2 reflects the scores achieved after the observations in Table 1.

**Table 2:** Shows the scores achieved by the Primary Health Center in pre-6S implementation phase

Scores in category		Possible score points			% Score
		Max points	Max %	Observed %	
Sort	10	20	100	50	
Set in order	1	20	100	5	
Shine	0	20	100	0	**30**
Standardize	4	20	100	20	
Sustain	8	20	100	40	
Safety	13	20	100	65	
TOTAL	36	120	100	**30**	

Interpretation of the scores is followed as per internationally carried protocols which are, 90–100% is called Excellent, meaning world-class 6S implementation. 75–89% scores if achieved it falls in good category, well-implemented, few improvements needed, 60–74% is called Fair – improvements required in key areas, 40–59% falls under Poor work atmosphere where major improvements are needed, and < 40% comes under critical where immediate corrective actions is needed. Our PHC falls under critical area.

### Observation summary

#### Sort (Organization)

Sort practices are inconsistent. While inventory and equipment are well-managed, walkways and safety hazards need urgent corrective action.

#### Set in order (Orderliness)

Orderliness is very poor. Visual management tools and employee training are urgently needed to improve spatial organization.

#### Shine (Cleanliness)

Cleanliness is critically poor. Immediate and structured cleaning protocols are needed across the facility.

#### StandardizE (Adherence)

Standardization efforts are insufficient. Visual tools and SOPs need to be implemented and reinforced throughout the workplace.

#### Sustain (Self-Discipline)

Sustainability is partial. Training is strong, but follow-through (audits, inspections, updated tools) is lacking.

#### Safety

Safety compliance is generally good, but environmental conditions and tool storage can be improved.

- Improvement suggestions given to the team of nurses for second phase implementation:
- Immediate Priorities: Cleanliness (Shine), Obstruction Hazards (Sort), and Workplace Organization (Set in Order).
- Quick Wins: Introduce labeling, cleaning schedules, and checklist formats.
- Long-Term Action: Reinforce 6S culture through leadership support, visual management tools, and weekly audits.

During the third phase observations were made by author in third month, after the staff implemented the changes, the findings have been collected. Table 3 shows the data collected during this phase of the study. The researcher observations were tabulated at various time intervals, on different days of the week, different shifts. The suggestions after the evaluation have been suggested later. Table 4 shows the scores achieved in Phase 3 of the study.

**Table 3:** Table showing Post-6S training and implementation evaluation scores of Primary Health Center

6S Check list used DATE: 01/08/2024 to 30/08/2024		Number of Problems		Rating				
		5 or more		0				
		3 to 4		1				
		2		2				
		1		3				
		None		4				
**Rating**								
		**0**	**1**	**2**	**3**	**4**	**Score**	
**Category**	**Item**							**Comments**
Sort (Organization)	Distinguish between what is needed and not needed						18	
	Unneeded equipment, tools, furniture, etc. are present					4		Workspace is clear of redundant items—excellent maintenance of organization
	Unneeded items are on walls, bulletin boards, etc					4		Only essential and updated materials displayed—very well managed
	Items are present in aisleways, stairways, corners, etc					4		All walkways are clutter-free, allowing safe and efficient movement
	Unneeded inventory, supplies, parts, or materials are present					4		No excess stock found. Inventory control is strong
	Safety hazards (water, oil, chemicals, machines) exist			2				Minor safety risks noticed. Recommend hazard signage or mitigation (e.g., wet floors or exposed wires)
Set in Order (Orderliness)	**A place for everything and everything in its place**						20	
	Correct places for items are not obvious					4		Clearly marked locations and standardized storage evident
	Items are not in there correct places					4		High discipline in placing tools/equipment back in designated spots
	Aisleways, workstations, equipment locations are not indicated					4		All stations and equipment zones are labeled and easy to identify
	Items are not put away immediately after use					4		Excellent habit observed—minimal idle or misplaced tools
	Height and quantity limits are not obvious					4		Limits well-marked; helps avoid overstocking or misplacement
Shine (Cleanliness)	**Cleaning, and looking for ways to keep it clean and organized**						19	
	Floors, walls, stairs, and surfaces are not free of dirt, oil and grease					4		Visibly clean and well-maintained environment
	Equipment is not kept clean and free of dirt, oil, and grease					4		Machinery is clean and ready for use—reflects good maintenance culture
	Cleaning materials are not easily accessible					4		Proper placement of cleaning tools—easily reachable
	Lines, labels, signs, etc. are not clean and unbroken					4		All signs are clean, intact, and readable
	Other cleaning problems (of any kind)are present				3			Minor dust/debris in corners or non-critical zones. Periodic deep cleaning advised
Standardize(Adherence)	**Maintain and monitor the first three categories**						20	
	Necessary information is not available					4		SOPs and instructions clearly posted and accessible
	All standards are not known and visible					4		Staff aware of operational norms—good signage use
	Checklists don't exist for all cleaning and maintenance jobs					4		All activities tracked via standardized formats
	All quantities and limits are not easily recognizable					4		Visual indicators effectively used (e.g., color codes, bins)
	Many items cant be located in 30 s					4		Quick access to items—layout highly functional
Sustain (Self-Discipline)	**Stick to the rules**						19	
	How many workers have not had 5'S training					4		All staff trained and aware of 6S roles/responsibilities
	Performance of 6 S audit weekly once					4		Regular audits conducted and recorded systematically
	Personal belongings not neatly stored					4		Workstations kept tidy with dedicated storage zones
	The number of times job aids are not available or up to date				3			Slight delays in updating few aids. Recommend scheduled reviews
	Inspections of 6S audit weekly once					4		Consistent inspection practices in place
Safety							18	
	Are employees wearing suitable PPE required for their current work activity					4		Full compliance observed—proper gear worn for all tasks
	Walkways, access to safety equipment is clearly identified and unobstructed (hazards, obstacles)					4		Emergency and safety zones clearly marked and accessible
	Is the working environment suitable for the work in hand (lighting, air quality, temperature)			2				Slight concerns (e.g., lighting or ventilation in certain zones). Improvement needed in facility conditions
	Are correct equipment/tools provided for the current work activity					4		Appropriate tools provided—no mismatches seen
	Tools, equipment, parts, WIP and PPE stored correctly and safely (appropriate height, location)					4		All items safely stored—no risk from falling or exposure
	TOTAL						114

**Table 4:** Scores achieved in Phase 3 of study, post 6S implementation

	Scores in category	Possible score points			
		Max points	Max %	Observed %	% Score achieved
Sort	18	20	100	90	**95%**
Set in order	20	20	100	100	
Shine	19	20	100	95	
Standardize	20	20	100	100	
Sustain	19	20	100	95	
Safety	18	20	100	90	
TOTAL	114	120	100	**95**	

#### General observations

Strengths: Set in Order, Standardize, Shine. Opportunities: Slight enhancements in safety and job aid updating processes. Actionable Recommendations: Improve facility lighting/air quality. Update job aids and hazard reporting more frequently. Conduct refresher audits for safety hazard spotting.

Interpretation of Results: SORT (Organization): Before: Visual clutter, unnecessary items, and some safety hazards. After: Exceptional organization, no unneeded items, clear and safe workspaces. Impact: + 40% increase, shifted from moderate to world-class performance.

#### Set in order (Orderliness)

Before: Disorganized, no visual markers or designated places. After: Complete transformation-everything has a place and is in place. Impact: + 95% jump, from disarray to model of visual control and workflow efficiency.

#### Shine (Cleanliness)

Before: Critically poor hygiene, no cleaning routines or accessible supplies. After: High cleanliness maintained across surfaces, tools, and zones. Impact: + 95% gain, reflecting a major cultural shift toward cleanliness and pride in the workspace.

#### Standardize (Consistency)

Before: Lack of SOPs, inconsistent practices, and poor visibility of standards. After: SOPs displayed, visual controls enforced, everyone aligned. Impact: + 80%, marking a huge leap in process discipline and efficiency.

#### Sustain (Discipline)

Before: Weak audit systems, some training gaps, unorganized personal storage. After: Consistent audits, full training coverage, and well-maintained personal areas. Impact: + 55%, showing embedded discipline and habit formation.

#### Safety

Before: Basic compliance with gaps in PPE use and environmental conditions. After: Proactive safety measures, well-identified hazards, strong compliance. Impact: + 25%, cementing a culture of safety consciousness.

The following recommendations were given for sustainability to the PHC Medical officer:

- Regular Refreshers: Continue training cycles and weekly audits to prevent regression.
- Visual Controls: Maintain labeling, SOPs, and feedback loops.
- Employee Engagement: Encourage ownership by involving staff in continuous 6S improvement ideas.
- Performance Dashboards: Use real-time 6S dashboards to track metrics visibly in the workspace.

**Fig. 1:**
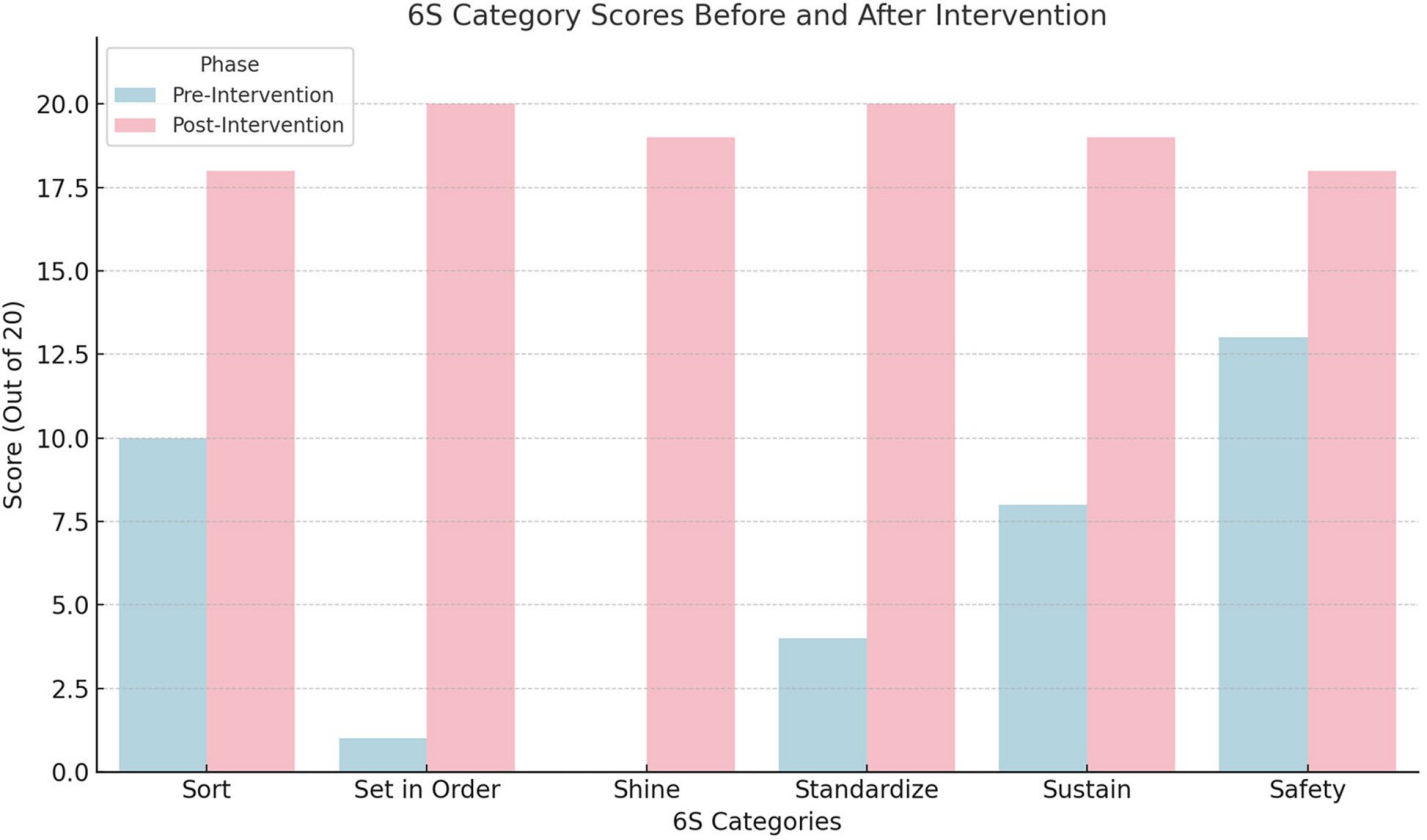
Parametric 6S domain score before and after intervention

**Fig. 2:**
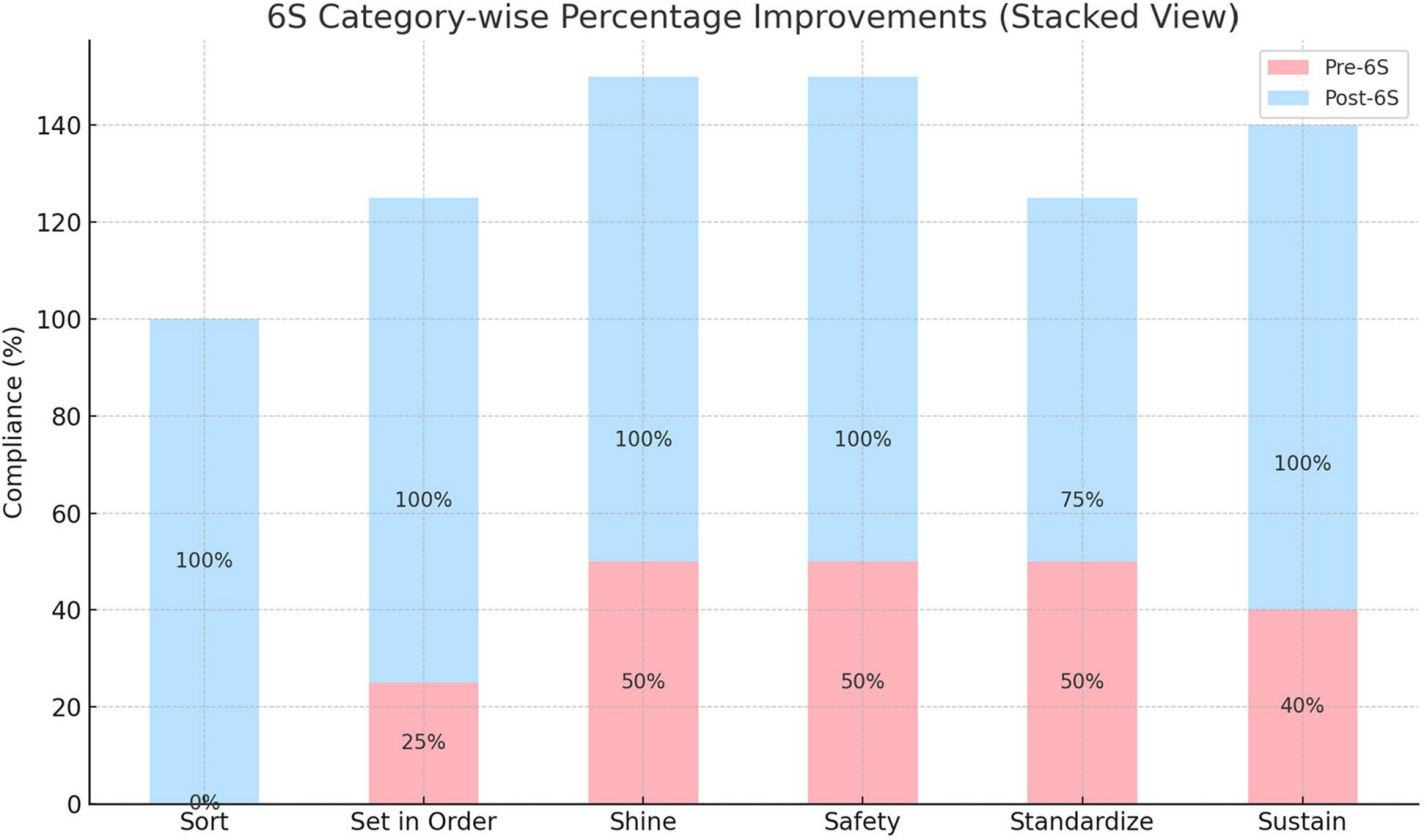
Stacked view of 6S category-wise percentage improvements

**Fig. 3:**
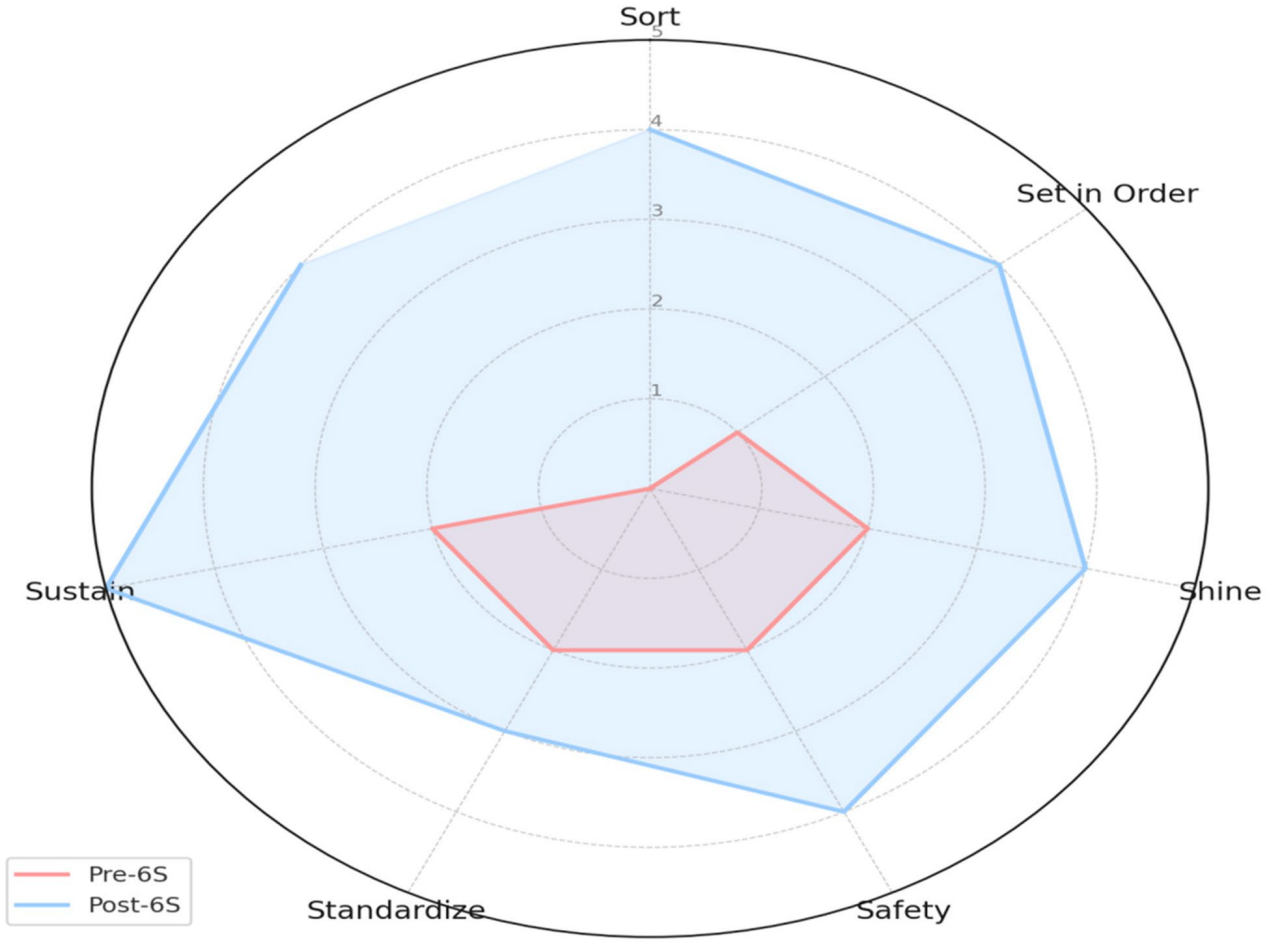
Spider Chart showing Pre versus Post 6S implementation of Key Performance Indicator comparison

**Fig. 4:**
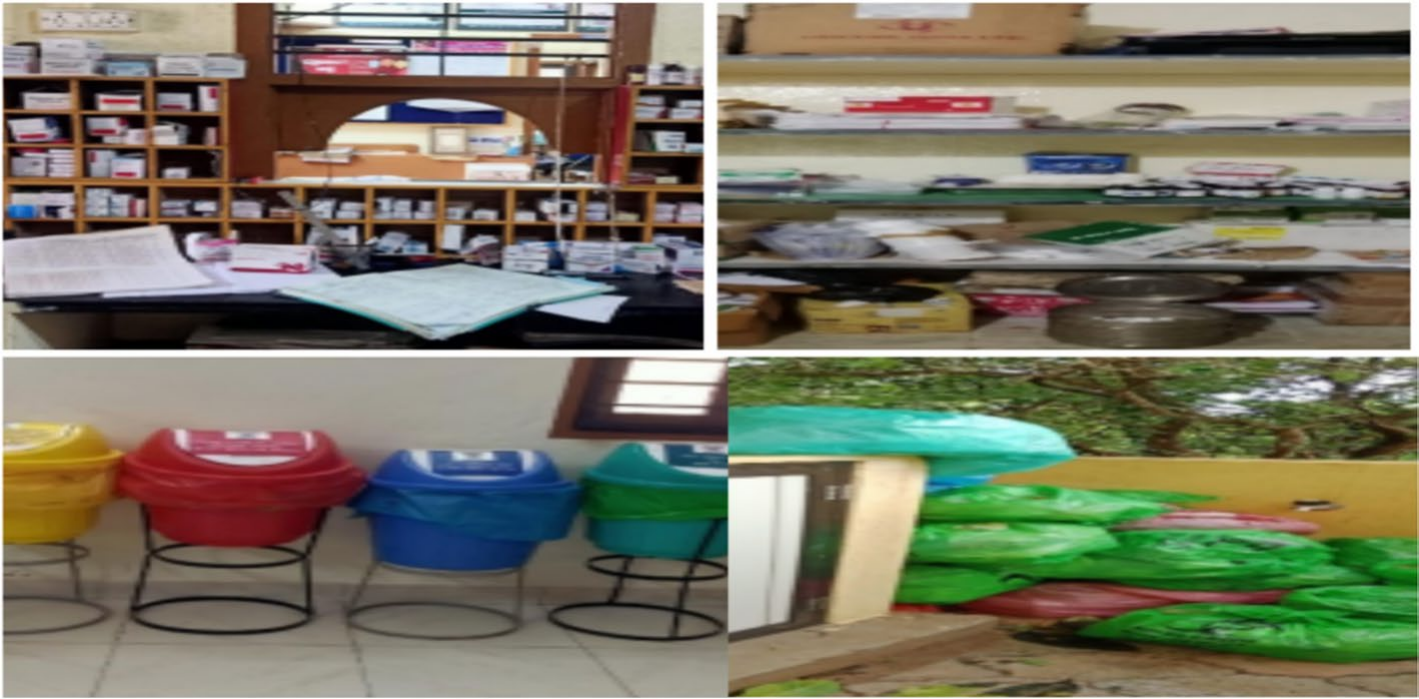
Photos before 6S implementation

**Fig. 5:**
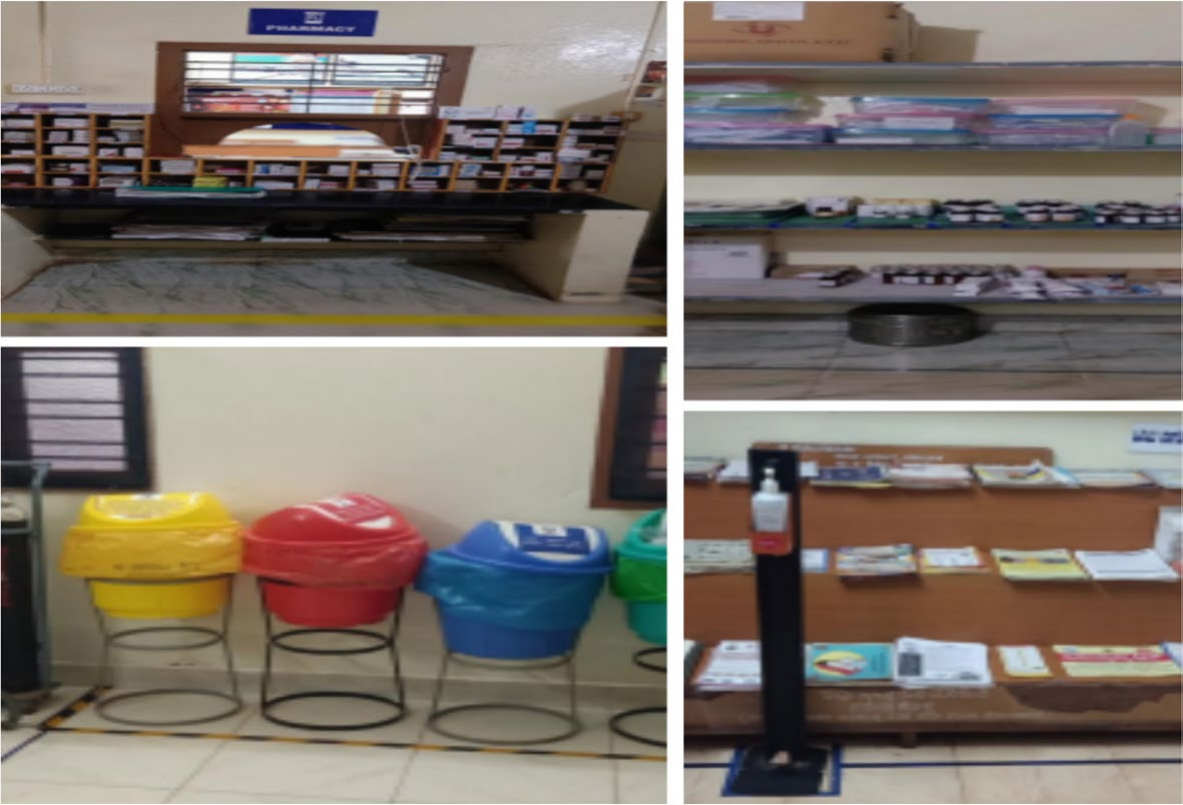
Photos after 6S implementtion

The pre- and post-intervention score across six 6S domains using both parametric and non-parametric tests showed in Figs. 1 and 2, with Paired Samples t-Test: t = −5.40, *p* = 0.0029, Statistically significant at *p* < 0.05 — confirms a strong improvement post-intervention. Chart 3 shows a figurative comparison between key performance indicators before and after intervention of 6S (Figs. 1, 2, 3, 4 and 5).

## Discussion

The implementation of the 6S methodology at a rural Primary Health Centre (PHC) demonstrated its strong potential as a transformative quality improvement strategy, even in low-resource healthcare environments.

Narayanamurthy et al. [1] and Suman & Prajapati [2] identified operational fragmentation and lack of standardized practices as major barriers in adopting Lean principles in India. Similarly, Bhat et al. [3] and Swarnakar et al. [7] noted that public sector facilities often lack the baseline discipline required to implement process optimization frameworks such as 6S.

Despite this, feasibility was established through participatory training, simplified toolkits, and structured rollout. As described by Singh et al. [6], Ajmera & Jain [4], and Kanamori et al. [12], training frontline staff, using color-coded bins, SOP charts, and audit checklists can empower teams even in peripheral facilities. The transformation at the PHC echoed the practical strategies suggested by Vaishnavi & Suresh [10, 13] for Lean readiness.

Domain-Specific Improvements and Literature Correlation: The post-intervention audit showed an overall improvement to 95%, with the most dramatic changes seen in:

Shine (0% → 95%): This confirmed enhanced environmental hygiene and routine cleaning protocols. Singh et al. [14] and Kanamori et al. [12, 15] showed similar outcomes in government labs and African health centers, emphasizing that regular cleaning schedules combined with staff responsibility rotation leads to enduring impact.

Set in Order (5% → 100%): The creation of logical workflows, labeled storage, and uncluttered pathways mirrored the interventions in Jiménez et al. [16] and Gijo & Antony [9], where workspace organization directly contributed to improved task efficiency and morale.

Standardize (20% → 100%) and Sustain (40% → 95%): These gains underline the effect of visual SOPs and daily checklists, a core lesson from studies by Parmar & Desai [17], Bhat et al. [5], and Samanta et al. [18], who emphasized that simple visual cues (floor markings, cleaning rotas, fire exits) can lock in compliance.

Sort (50% → 90%) and Safety (65% → 90%): Improvements in sorting and safety were reflected in better segregation of materials, reduction of trip hazards, and consistent PPE use. These changes align with prior findings from Rathore & Srivastava [19], Rathi et al. [20], and Isfahani et al. [21], who advocated for using structured layout zoning and routine safety briefings in lean deployments.

Category-wise Reflections:

- Sort: Prior to the intervention, none of the four indicators under the'Sort'pillar were satisfied. The space was cluttered, incomplete work was present, and no red-tag system was implemented. Post-6S, all criteria were fully met. This echoes findings from Kanamori et al. [12, 15, 22], who emphasized that initial decluttering lays the foundation for all subsequent improvements in Lean frameworks, especially in low-resource settings.
- Set in Order: Improvement from 1/4 to 4/4 reflects enhanced visual control and organization. Visual markings, item labeling, and clear designation of spaces were major contributors, as also observed by Singh et al. [14] and Swarnakar et al. [7]. Improved order directly impacts operational efficiency and task clarity, particularly beneficial in PHCs where multitasking is common.
- Shine: The increase from 2/4 to 4/4 in this domain supports the conclusion that workplace cleanliness is not only feasible but also sustainable when standard procedures are institutionalized. Similar success was reported by Singh MG et al. [14] in a government laboratory in India and aligns with Gijo & Antony [21] who demonstrated improvements in outpatient areas using Lean tools.
- Safety: A significant change from 2/4 to 4/4 was observed. Fire exits, extinguishers, MSDS, and emergency protocols became clearly posted and functional. This directly aligns with Antony et al. [23] and Rathi et al. [20], who found that visual safety tools and regular reinforcement improved preparedness and reduced workplace hazards.
- Standardize: While this domain improved from 2/4 to 3/4, full paperless standardization remains a challenge. The missing indicator suggests systemic barriers such as digital literacy, resource availability, or workflow resistance. This mirrors findings by Vaishnavi and Suresh [13] who emphasized that standardization often requires longer-term reinforcement and digital integration to be sustainable.
- Sustain: Perhaps most impressively, this domain jumped from 2/5 to 5/5. Regular procedures were followed, boards were updated, and cleanliness was maintained. These results affirm the global evidence that sustained Lean benefits require cultural buy-in, not just procedural fixes [24, 25].

The transformation documented aligns with systematic reviews showing Lean’s adaptability across varying healthcare contexts [3, 18, 23, 26]. The improvements in workplace order and safety reflect what Antony et al. [25] found in NHS settings and Jiménez et al. [16] in extending 5S into 6S with a focus on safety. Moreover, the human-centric, participatory method—driven by on-site observations and staff involvement—reaffirms the views of Rathi et al. [20] and Samanta et al. [18], who emphasized engagement as the cornerstone of Lean success.

### Implications

The fact that such profound improvements occurred in a PHC without high financial investments is significant. It demonstrates that quality improvement tools like 6S are not only technically applicable, but culturally acceptable in India’s public health infrastructure. These insights could inform national scale-up strategies and training modules for PHCs and district hospitals under initiatives like Kayakalp.

### Behavioral transformation and staff engagement

Beyond quantitative data, qualitative changes were visible in team behavior and accountability. Staff began to internalize order and cleanliness as integral to patient care, not just a compliance requirement. This aligns with Rathore & Srivastava [19], who emphasized mindset shift as the most enduring output of Lean.

The introduction of a “6S champion” model, wherein staff rotated audit responsibilities, increased ownership and reduced dependency on external enforcement—a strategy echoed in Kanamori et al. [22] and Maqbool et al. [27]. The human-centered, low-cost design of the intervention ensured inclusivity and adaptability, which are essential for success in government PHCs, as emphasized by Varkey & Kollengode [28].

### Comparison with national and global studies

While 6S has been extensively documented in tertiary care [21, 29], laboratories [14, 30], and pharmaceutical workflow [31, 32], this study contributes uniquely by demonstrating its success in a rural primary care context.

Our pre-post leap (from 30 to 95% in workplace audit, and 36% to 96% in compliance) surpasses many existing Indian case studies in magnitude, despite being implemented in a more constrained setting. This reinforces the findings from Rathi et al. [20, 26], who argued that when appropriately localized, Lean strategies yield exponential benefits even at the lowest rung of the care ladder.

### Implications for health policy and quality assurance

This study provides a scalable model that can align with India’s National Quality Assurance Standards (NQAS) and the Kayakalp program. By aligning 6S practices with national health goals, PHCs can bridge the gap between accreditation standards and ground realities.

#### Limitations

While this study demonstrated significant improvements through the implementation of the 6S methodology, several limitations must be acknowledged:

Single-site design: The study was conducted in one rural PHC, which limits generalizability. Broader implementation across diverse healthcare settings is necessary to confirm scalability. Short-term follow-up: Post-intervention assessments were done soon after implementation. Longer-term studies are required to assess sustainability and detect any regression to previous practices. Non-randomized design: The absence of a control group means improvements cannot be wholly isolated from external influences such as seasonal variations or unrelated training programs. Observer bias: Since internal teams were involved in assessment, there is a potential risk of observer or Hawthorne bias. However, this was mitigated through structured tools and repeat validations. No cost analysis: Although Lean interventions are low-cost by design, the study did not conduct a formal economic evaluation to compare savings from efficiency versus resource investment. Lack of patient-centered metrics: While the study focused on workplace organization and staff compliance, it did not measure patient satisfaction, waiting times, or clinical outcomes.

## Conclusion

This study affirms that the 6S methodology is not only feasible but also highly effective in improving the operational standards of a resource-constrained Primary Health Centre (PHC) in India. Post-implementation, the workplace audit score increased from 30 to 95%, and binary compliance improved from 36 to 96% changes that were statistically validated using Wilcoxon Signed-Rank and McNemar’s tests.

The inclusion of"Safety"as a sixth domain proved especially critical in enhancing infection control practices and biomedical waste management. Equally important was the human-centric approach: training sessions, visual reminders, peer champions, and audit feedback fostered intrinsic motivation and team ownership—critical to sustaining change.

Importantly, this study demonstrates that Lean tools like 6S can be implemented at low-cost, scalable, and context-sensitive, offering a viable pathway to strengthen India’s public health infrastructure from the bottom up. As echoed in prior research, the key to sustained impact lies in institutionalizing 6S through regular training, visual dashboards, and periodic audits.

## Supplementary Information

Supplementary Material 1

## Data Availability

All data generated or analysed during this study are included in this published article.

## Abbreviations

6S: Six steps of 6S
PHC: Primary health center
LSS: Lean Six Sigma
FISM: Fuzzy interpretive structural modeling
SOP: Standard Operating Procedures
NHS: National Health System
NQAS: National Quality Assurance Standards
